# Identification of prognostic biomarkers associated with stromal cell infiltration in muscle‐invasive bladder cancer by bioinformatics analyses

**DOI:** 10.1002/cam4.3372

**Published:** 2020-08-12

**Authors:** Pan Li, Jinlong Cao, Jianpeng Li, Zhiqiang Yao, Dali Han, Lijun Ying, Zhiping Wang, Junqiang Tian

**Affiliations:** ^1^ Department of Urology Lanzhou University Second Hospital Lanzhou China; ^2^ Key Laboratory of Gansu Province for Urological Diseases Lanzhou China; ^3^ Clinical Center of Gansu Province for Nephron‐urology Lanzhou China; ^4^ The Second Clinical Medical College Lanzhou University Lanzhou China

**Keywords:** bioinformatics, hub genes, muscle‐invasive bladder cancer, stromal cells, tumor microenvironment

## Abstract

Muscle‐invasive bladder cancer (MIBC) is one of the common malignant tumors. Patients with MIBC still have high tumor recurrence and progression rates after surgery. Bioinformatics analysis of stromal infiltration‐related genes in the tumor microenvironment (TME) of MIBC patients was performed in this study to determine the major stromal cells types and biomarkers for their progression and poor prognosis. The ESTIMATE algorithm was applied to evaluate the stromal score and immune score of samples from MIBC patients in The Cancer Genome Atlas (TCGA) and found that stromal score was closely related to the clinical characteristics of the patients. The Gene Set Enrichment Analysis (GSEA) further revealed that stromal cells were involved in biological processes such as activation of leukocytes and positive regulation of cell migration during MIBC progression, as well as PI3K‐Akt, MAPK, and Rap1 signaling pathways. Five hub genes related to prognosis, including *ACTA2, COL5A1, DCN, LUM*, and *PRRX1* were identified by the Weighted Gene Co‐Expression Network Analysis (WGCNA), Protein‐Protein Interaction (PPI), survival analysis, and Oncomine, Gene Expression Omnibus (GEO) database validation. Besides, we identified five stromal cell types associated with overall survival time, among which chondrocytes and fibroblasts were identified as the major stromal cell types through correlation analysis. Finally, the Receiver Operating Characteristic (ROC) curve and immunohistochemistry were used to verify the diagnostic value and expression of hub genes in different invasive tumors. In summary, we investigated the biological behavior of stromal cells in the TME of MIBC to promote tumor progression obtained hub genes associated with progression and poor prognosis and identified the main stromal cells types in the TME.

## Graphical abstract

Through our analysis, we found that stromal cells are closely related to the clinical prognosis of patients. Stromal cells are mainly involved in cell migration, leukocyte activation, and PI3K/AKT, MAPK, and Rap1 signaling pathways in tumor progression. In addition, we identified the hub genes of stromal cells promoting tumor progression based on bioinformatics analysis, and further identified chondrocytes and fibroblasts as the main types of 13 stromal cells.

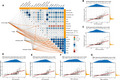

## 1 INTRODUCTION

Bladder cancer (BC) is the 10th most common tumor in the world, with 549 000 new cases and 200 000 deaths worldwide in 2018.^1^ According to the degree of invasion of the muscular layer, it is divided into nonmuscle‐invasive bladder cancer (NMIBC) and muscle‐invasive bladder cancer (MIBC). Compared with NMIBC, MIBC is more likely to progress, and its clinical prognosis is worse.^2^, ^3^ Although radical cystectomy is the main method for the treatment of MIBC, the postoperative 5‐year recurrence‐free survival rate is still low in patients with high T stage (The patients with pT_4_ is 36%).^4^ Postoperative tumor recurrence and progression are still the main problems in the treatment of MIBC. Neoadjuvant chemotherapy, radiotherapy, and immunotherapy can reduce the possibility of recurrence and progression after MIBC surgery, however, they all have different degrees of adverse reactions.^3^ Moreover, the key to the treatment of MIBC is to develop a systematic treatment plan for different mechanisms of tumor progression and recurrence. Thus, it is particularly important to study the different mechanisms of MIBC progress to improve the prognosis of patients.

The cellular environment in which tumor cells are located is called the tumor microenvironment (TME), which is mainly composed of blood vessels, lymphatic vessels, extracellular matrix (ECM), stromal cells, immune/inflammatory cells, secreted proteins, RNA, and small organelles, affecting the biological function of tumor cells.^5^ When the proliferation and growth of tumor cells are uncontrolled, cell hypoxia, oxidative stress, and acidosis appear in TME, which cause the adjustment of ECM, lead to the response of adjacent stromal cells and immune cells, induce angiogenesis, and finally lead to tumor metastasis.^6^ Stromal cells are an important part of TME, which can provide growth signals and intermediate metabolites for the growth of a variety of tumor cells, and can inhibit or promote the metastasis of tumor cells, but the overall function of stromal cells is more conducive to the survival and transfer of tumor cells to provide a suitable environment for its progress and transfer.^7^, ^8^, ^9^ In the course of antitumor treatment, stromal cells also enhance the resistance of tumor cells to treatment.^10^ Related studies^11^ have shown that immune cells in the bladder TME can promote the occurrence and development of MIBC, but the relationship between stromal cells as another important cell in the TME and the progress of MIBC has not yet been determined.

In this study, we aimed to use the ESTIMATE scoring method to perform stromal score on the RNA‐Seq gene expression profiles data of MIBC from The Cancer Genome Atlas (TCGA) database.^12^ To determine the stromal score relationship with clinical characteristics of patients and verify the relationship between stromal cells and MIBC progression. Finally, we found that stromal cells mainly regulate the progress of MIBC by participating in biological processes such as cell migration, leukocyte activation, and signaling pathways such as PI3K‐Akt, MAPK, and Rap1. Five hub genes associated with stromal cells and five types of stromal cells significantly associated with prognosis were identified. Our study investigated the effect of stromal cells on MIBC aggressivity and provided a basis for exploring new molecular targeted therapies to improve the prognosis of MIBC.

## 2 MATERIALS AND METHODS

### 2.1 Data sources

**FIGURE 1:**
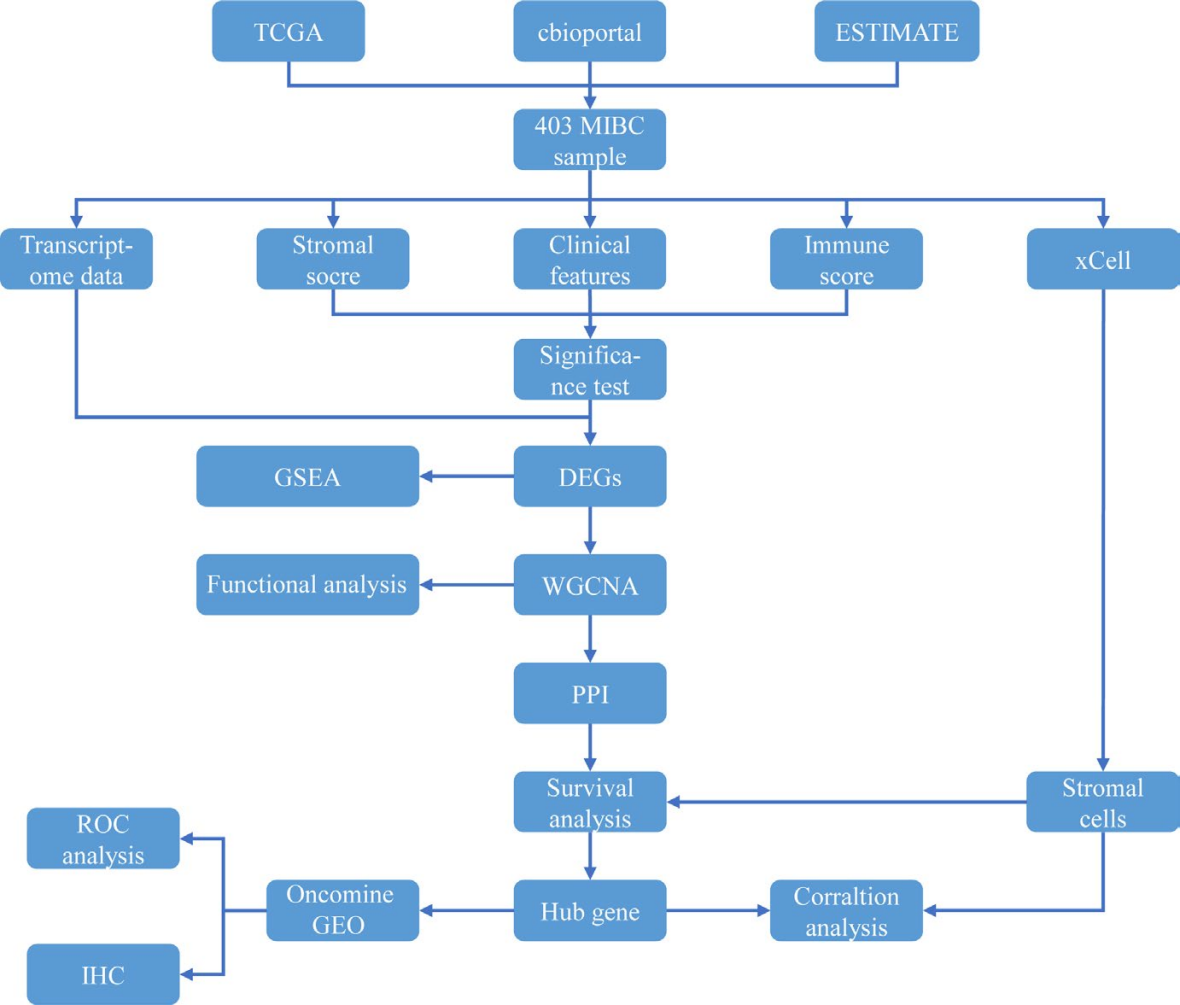
The workflow of the selection process for the eligible studies in the analysis. DEGs, differentially expressed genes; GEO, Gene Expression Omnibus; GSEA, Gene Set Enrichment Analysis; IHC, immunohistochemical; MIBC, muscle‐invasive bladder cancer; PPI, Protein‐ Protein Interaction; ROC, Receiver Operating Characteristic; TCGA, The Cancer Genome Atlas; WGCNA, Weighted Gene Co‐Expression Network Analysis.

Data were obtained from the following approaches: (a) RNA‐Seq gene expression profiles data of BC patients from the TCGA database (http://portal.gdc.cancer.gov). (b) Clinical information (including gender, age, Tumor‐Node‐Metastasis (TNM) stage, clinical stage, pathological grade, tumor pathology subtype, overall survival time) from cbioportal (http://www.cbioportal.org). (c) BC sample stromal score and the immune score of the patient from ESTIMATE (http://bioinformatics.mdanderson.org/estimate). The exclusion criteria were as follows: (a) NMIBC tissue sample. (b) Multiple tissue samples corresponding to the same patient. (c) Adjacent cancer tissue samples. (d) Nonbladder primary tumor tissue samples. (e) The transcriptome sequencing data, clinical information, and stromal and immune scores of the samples were incomplete. Finally, 403 samples were included. A dataset of the gene expression profile of accession number GSE31684 was obtained from the Gene Expression Omnibus (GEO) database (https://www.ncbi.nlm.nih.gov/geo), and 78 patients from MIBC were included according to T staging. The workflow is displayed in Figure 1.

### 2.2 Differentially expressed genes (DEGs) screening

In R 3.6.1 (R Foundation for Statistical Computing, Vienna, Austria), the optimal cutoff values for overall survival analysis of stromal score by maxstat package was used as the grouping criterion.^13^ Sequencing data were normalized and analyzed for differences. In order to reduce the false‐positive rate, log_2_ |fold change (FC)| was corrected by the “ashr” method.^14^ |FC| > 1.5 and *P* < .05 were used as the screening criteria for DEGs.

### 2.3 Gene set enrichment analysis (GSEA)

The GSEA is a calculation method to determine whether the prior gene set is statistically significant in the studied gene set.^15^ The GSEA was performed to find enriched terms predicted to have a correlation with the stromal score by using the clusterProfiler package.^16^ The parameter settings were setSize > 100, nPerm = 1000 and *P* < .05. The first four items were selected as key biological functions and pathways by the value of *P*.

### 2.4 Weighted gene co‐expression network analysis (WGCNA)

The WGCNA can find co‐expressed gene modules, explore the association between gene networks and clinical phenotypes, and core genes in the network.^17^ DEGs were used to construct a gene co‐expression network using the WGCNA package, and the relationship between modules and clinical phenotypes was analyzed.^18^ Module and phenotype associated genes were screened under the condition of gene significance (GS) >0.2 and module membership (MM) >0.8. The genes related to clinical progression were obtained by taking the intersection of genes related to different clinical phenotypes.

### 2.5 Functional analysis

Gene Ontology (GO) analysis is to describe the biological and molecular functions of genes and to describe different levels and dimensions of cellular components. Kyoto Encyclopedia of Genes and Genomes (KEGG) pathway analysis can bring together genes that are highly similar in sequence and perform the same function. DAVID (https://david.ncifcrf.gov) provides a comprehensive set of functional annotation tools for investigators to understand biological meaning behind large list of genes.^19^ DEGs in the key module were put into DAVID for GO and KEGG analysis. *P* < .05 was set as the screening criteria for analysis results.

### 2.6 Construction of Protein‐Protein Interaction (PPI) network

The genes related to clinical progression were imported into the STRING online database (https://string‐db.org), and a PPI network was constructed with a mutual score greater than 0.4 as a threshold.^20^ The plug‐in in Cytoscape v3.6.1 was used to screen the top 30 genes in the network as the central genes using five algorithms: betweenness, closeness, degree, Edge Percolated Component (EPC), and Maximal Clique Centrality (MCC).^21^, ^22^ The genes obtained by various algorithms were intersected to obtain the hub genes.

### 2.7 Overall survival analysis of hub genes and stromal cells

The xCell is a R package that performs cell type enrichment analysis from gene expression data for 13 kinds of stromal cells in TME.^23^ The samples were divided into high and low groups according to the median enrichment score of stromal cells and median expression of hub genes. GraphPad Prism 8 (GraphPad Prism Software Inc, San Diego, California) was used to analyze the difference in overall survival between high and low group samples. *P* < .05 was a significant difference in overall survival time.

### 2.8 Correlation analysis between hub genes and stromal cells

The heat map of enrichment score of stromal cells, hub genes expression, and clinical phenotype correlation was drawn by the ggcor package. Using ggstatsplot package to draw scatter plot, cor > 0.6 and *P* < .001 indicates that there was a strong correlation between the two.

### 2.9 Verify the expression of hub genes related to clinical progression

The Oncomine database was used to verify hub genes expression differences in infiltrating BC and superficial BC. The expression of hub genes in high pathological stage (T_3‐4_) and low pathological stage (T_2_) MIBC was further verified in the GSE31684 gene expression profile, *P* < .05 was used as the screening criterion for the differences between different pathological levels. The Receiver Operating Characteristic Curve (ROC) curve was used to evaluate the diagnostic role of hub genes in high and low grade MIBC. The area under the curve (AUC) >0.6 and *P* < .01 were used as the diagnostic criteria. Based on the results of THE HUMAN PROTEIN ATLAS (https://www.proteinatlas.org) immunohistochemistry, the expression levels of hub genes in MIBC at different pathological levels were verified.

## 3 RESULTS

### 3.1 Stromal score was significantly correlated with clinical phenotype and overall survival outcome

**FIGURE 2:**
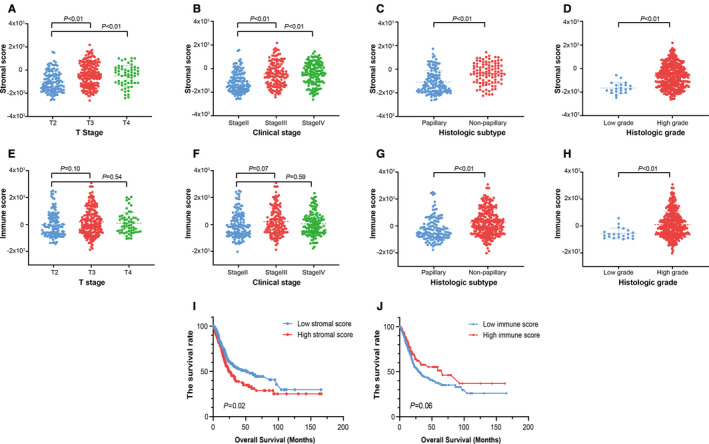
Stromal and immune scores were significantly associated with clinical phenotypes. A‐D, The distribution of stromal scores in different T stage, clinical stage, histologic subtype, and histologic grade. E‐H, The distribution of immune scores in different T stage, clinical stage, histologic subtype, histologic grade. I, Best survival analysis based on stromal score grouping. J, Best survival analysis based on immune score grouping

Through the significance test of stromal and immune score of patients with different clinical information. It is found that there were significant differences in the stromal score (*P* < .05) between low T stage (T_2_) and high T stage (T_3‐4_) MIBC and low clinical stage (stage II) and high clinical stage (stage III‐IV) MIBC. There were significant differences in the stromal and immune score of papillary and nonpapillary carcinomas, low grade and high grade carcinomas (*P* < .05) (Figure 2A‐H). The maxstat package in R software was used to obtain the optimal survival threshold of the stromal and immune score and survival analysis. The results showed that the overall survival time of patients with the low stromal score was higher than that of the high stromal score (*P* < .05). There was no significant difference between the high and low immune score and the overall survival time of patients (*P* > .05) (Figure 2I‐J).

### 3.2 Identification of DEGs associated with the stromal score

**FIGURE 3:**
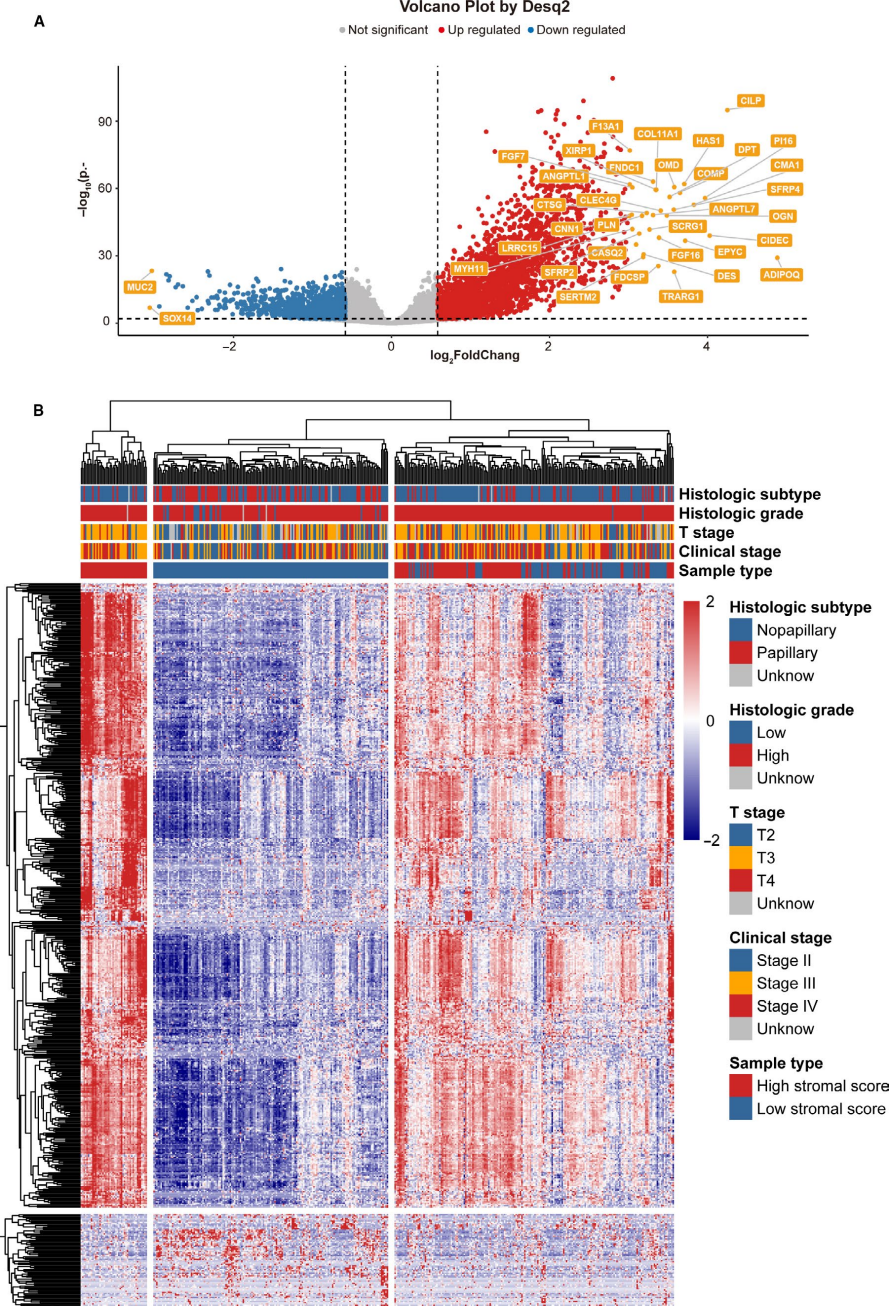
Volcano plot and heatmap by Desq2 analysis result. A, Volcano plot of the DEGs. The blue dots represent downregulated DEGs. The red dots represent upregulated DEGs. The orange label indicates DEGs that the log_2_|FC|> 3. B, Heatmap of some of the significantly DEGs. DEGs, differentially expressed genes

The optimal cutoff values for the survival analysis was used to group high and low stromal score samples to obtain 258 patients with the low stromal score and 145 patients with the high stromal score. The clinical information was shown in Table 1. After screening the differential genes, a total of 3890 DEGs were obtained, including 2661 upregulated genes and 1229 downregulated genes (Figure 3A). The distribution of the expression of some significantly DEGs in tissue samples is shown in Figure 3B.

**TABLE 1:** Clinical features of the patients with MIBC were divided into different stromal score

Characteristics		Group		χ	*P* value
		Low (n = 258) (%)	High (n = 145) (%)		
Age (Mean ± SD)		67.13 ± 11.32	69.90 ± 8.92	**—**	**.01**
Gender	Male	196 (75.97%)	102 (70.34%)	1.52	.22
	Female	62 (24.03%)	43 (29.66%)		
Clinical stage	Stage II	103 (39.92%)	26 (17.93%)	**23.50**	**3.2e^−5^**
	Stage III	82 (31.78%)	56 (38.62%)		
	Stage IV	71 (27.52%)	63 (43.45%)		
	Stage X	2 (0.78%)	0 (0.00%)		
Histologic grade	Low	21 (8.14%)	0 (0.00%)	**12.48**	**2.0e^−3^**
	High	235 (91.09%)	144 (99.31%)		
	Unknow	2 (0.78%)	1 (0.69%)		
Histologic subtype	Papillary	102 (39.53%)	28 (19.31%)	**17.39**	**1.67e^−4^**
	Nonpapillary	153 (59.30%)	115 (79.31%)		
	Unknow	3 (1.16%)	2 (1.38%)		
TNM staging system	T_2_	93 (36.05%)	26 (17.93%)	**36.47**	**5.97e^−8^**
	T_3_	103 (39.92%)	89 (61.38%)		
	T_4_	30 (11.63%)	28 (19.31%)		
	T_x_	32 (12.40%)	2 (1.38%)		
	N_0_	159 (61.63%)	75 (51.72%)	**16.76**	**2.30e^−4^**
	N_1‐3_	66 (25.58%)	63 (43.45%)		
	N_x_	33 (12.79%)	7 (4.83%)		
	M_0_	144 (55.81%)	48 (33.10%)	**19.20**	**6.80e^−5^**
	M_1_	6 (2.33%)	5 (3.45%)		
	M_x_	108 (41.86%)	92 (63.45%)		

Note

The bold values represent *P* < .05; The difference analysis was statistically significant.

### 3.3 Stromal cell‐related genes were mainly involved in biological functions and key pathways

**FIGURE 4:**
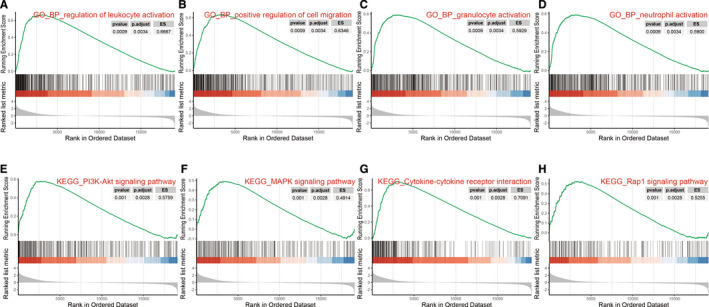
GSEA results of stromal cell‐related genes. A‐D, GSEA of stromal cell‐related genes in BP; (E‐H) GSEA of stromal cell‐related genes in KEGG pathway. BP, biological process; ES, enrichment score; GO, Gene Ontology; GSEA, gene set enrichment analysis; KEGG, Kyoto Encyclopedia of Genes and Genomes

GSEA was used to conduct biological function and key pathway enrichment analysis of high and low stromal score samples. Genes associated with stromal cells were mainly involved in biological processes such as regulating leukocyte activation, positive regulation of cell migration, granulocyte activation, and neutrophil activation (Figure 4A‐D). And they participated in PI3K‐Akt, MAPK, Rap1, and other signaling pathways and cytokine‐cytokine receptor interaction (Figure 4E‐H).

### 3.4 Identification of DEGs in key modules that were significantly related to clinical progress

**FIGURE 5:**
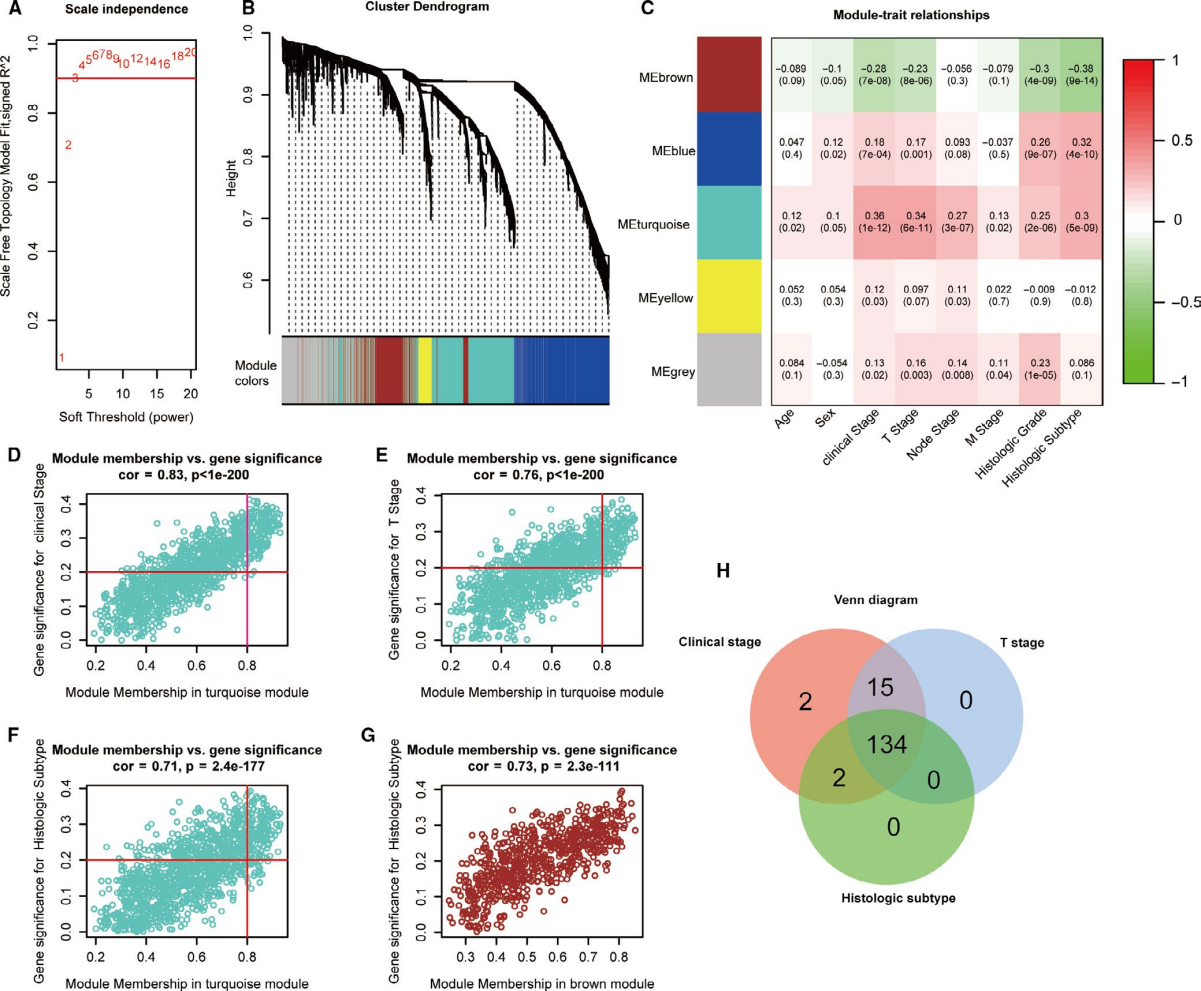
Construction of a weighted gene co‐expression network. A, Analysis of the scale‐free topology model fit index for soft threshold powers (β). B, A cluster dendrogram was built based on the dissimilarity of the topological overlap, which presents five gene co‐expression modules in MIBC, the grey module indicates none co‐expression between the genes. C, Heatmap of the correlation between module eigengenes and clinical traits of MIBC. D‐F, The scatterplot of GS for clinical stage, T stage and histologic subtype vs MM in the turquoise module. G, A scatterplot of GS for histologic subtype vs MM in brown module. H, Common DEGs with significantly related clinical phenotypes in the turquoise module. DEGs, differentially expressed genes; GS, gene significance; MIBC, muscle‐invasive bladder cancer; MM, module membership

Performing the WGCNA analysis of DEGs, the co‐expression network was consistent with the scale‐free network diagram when the network soft threshold was 3. The research results showed that DEGs could be divided into five modules (brown, blue, turquoise, yellow, grey) based on their overall functions, in which there were 663 DEGs in brown module, 1088 DEGs in blue module, 1152 DEGs in turquoise module, 160 DEGs in brown module and 827 DEGs in brown module, and the DEGs in the grey module were not included in other modules (Figure 5A‐C). Genes in the turquoise module were significantly related to phenotypes of MIBC such as clinical stage (cor = .83, *P* < 1e^−200^), pathological T stage (cor = .76, *P* < 1e^−200^), and histologic subtype (cor = .71, *P* = 2.4e^−177^) (Figure 5D‐G). The brown module was related to the histologic subtype phenotype (cor = 0.73, *P* = 2.3e^−111^). Obtained 134 common DEGs in the turquoise module with three significantly related clinical phenotypes (Figure 5H).

### 3.5 GO and KEGG analysis

The GO and KEGG pathway analysis of the turquoise and brown modules obtained by WGCNA found that the turquoise module was mainly enriched in the composition of the extracellular stromal (GO: 0 030 198, *P* = 2.62e^−35^) and participated in the focal adhesion pathway (hsa04510, *P* = 4.91e^−17^). The Brown module is mainly enriched in steroid metabolism biological processes (GO: 0 008 202, *P* = 4.53e^−05^) and participated in chemical carcinogenic pathways (hsa05204, *P* = 2.70e^−07^) (Table 2).

**TABLE 2:** The results for GO‐BP function and KEGG pathway enrichment analysis. (top 3 in each module are listed)

Module	GO_BP term	*P* value	KEGG_term	*P* value
Turquoise	GO:0030198~extracellular matrix organization	2.62e^−35^	hsa04510: focal adhesion	4.91e^−17^
	GO:0007155~cell adhesion	4.17e^−30^	hsa04512: ECM‐receptor interaction	1.80e^−16^
	GO:0030199~collagen fibril organization	8.25e^−19^	hsa04151: PI3K‐Akt signaling pathway	1.13e^−12^
Brown	GO:0008202~steroid metabolic process	4.53e^−05^	hsa05204: chemical carcinogenesis	2.70e^−07^
	GO:0019369~arachidonic acid metabolic process	9.28e^−05^	hsa00982: drug metabolism ‐ cytochrome p450	2.42e^−06^
	GO:0055114~oxidation‐reduction process	9.74e^−05^	hsa00980: metabolism of xenobiotics by cytochrome p450	3.61e^−05^

### 3.6 Identification of hub genes in the PPI network

**FIGURE 6:**
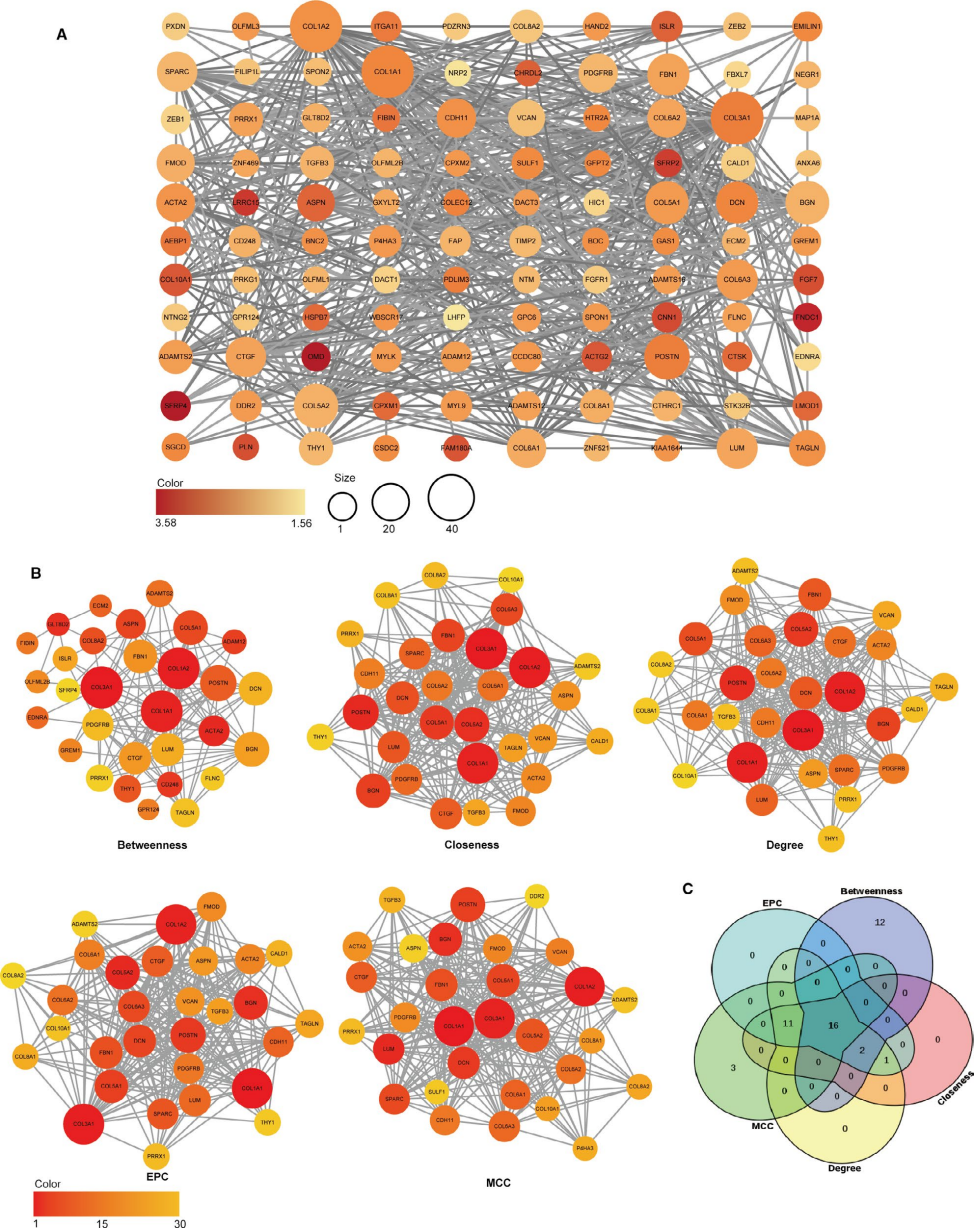
Construction of the PPI network. A, PPI network. The color of the nodes in the PPI network reflects log_2_ |FC| value, and the size of the nodes represents the number of proteins interacting with each other. B, Screening the top 30 genes in the PPI network as central genes using five algorithms including Betweenness, Closeness, Degree, EPC, and MCC. The color and size of the nodes in the protein‐protein interaction network reflects the score level of calculation methods. C, Various algorithms obtain a Venn diagram of a common gene. EPC, edge percolated component; MCC, maximal clique centrality; PPI, protein‐protein interaction

The 134 DEGs related to clinical progression obtained from WGCNA analysis were imported into the STRING database. The results were imported into Cytoscape 3.7.2 to calculate the network and the topological characteristics of each node. The PPI complex was filtered to obtain 110 nodes and 559 pairs of PPI relationships (Figure 6A). Based on five algorithms in the cytoHubba plug‐in, the protein interaction network consisting of the first 30 DEGs scored by each algorithm was selected. These genes were located at the central position of the protein interaction network, playing an important role in the occurrence and development of the disease (Figure 6B). The protein interaction network composed of five algorithms contained 16 common hub genes (*COL3A1, COL1A1, COL1A2, ACTA2, POSTN, LUM, BGN, COL5A1, DCN, ASPN, FBN1, COL8A2, CTGF, PDGFRB, ADAMTS2, PRRX1*) (Figure 6C).

### 3.7 Overall survival analysis of hub genes and stromal cells

**FIGURE 7:**
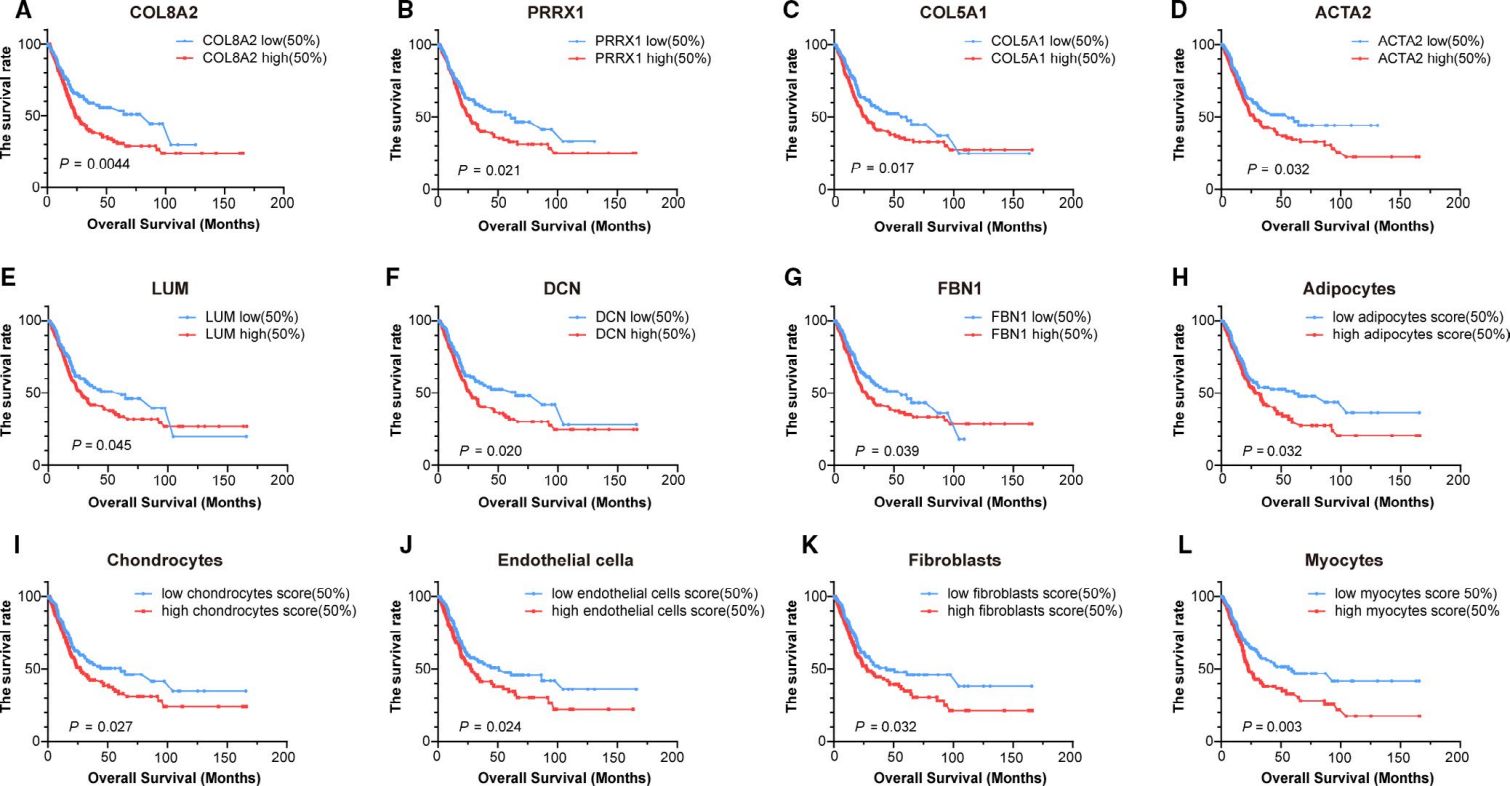
Kaplan‐Meier curves of the risk biomarkers and stromal cells of MIBC in TCGA. A‐G, Survival analysis of high and low expression of hub genes. H‐L, Survival analysis of high and low stromal cells score. MIBC, muscle‐invasive bladder cancer; TCGA, The Cancer Genome Atlas

The clinical information was used to analyze the overall survival time of hub genes and stromal cells. Seven hub genes (*ACTA2, COL8A2, COL5A1, DCN, FBN1, LUM, PRRX1*) expression levels and the enrichment scores of five stromal cells (adipocytes, chondrocytes, endothelial cells, fibroblasts, and myocytes) were negatively correlated with the overall survival time of patients (*P* < .05) (Figure 7).

### 3.8 The expression of hub genes was related to stromal cells

**FIGURE 8:**
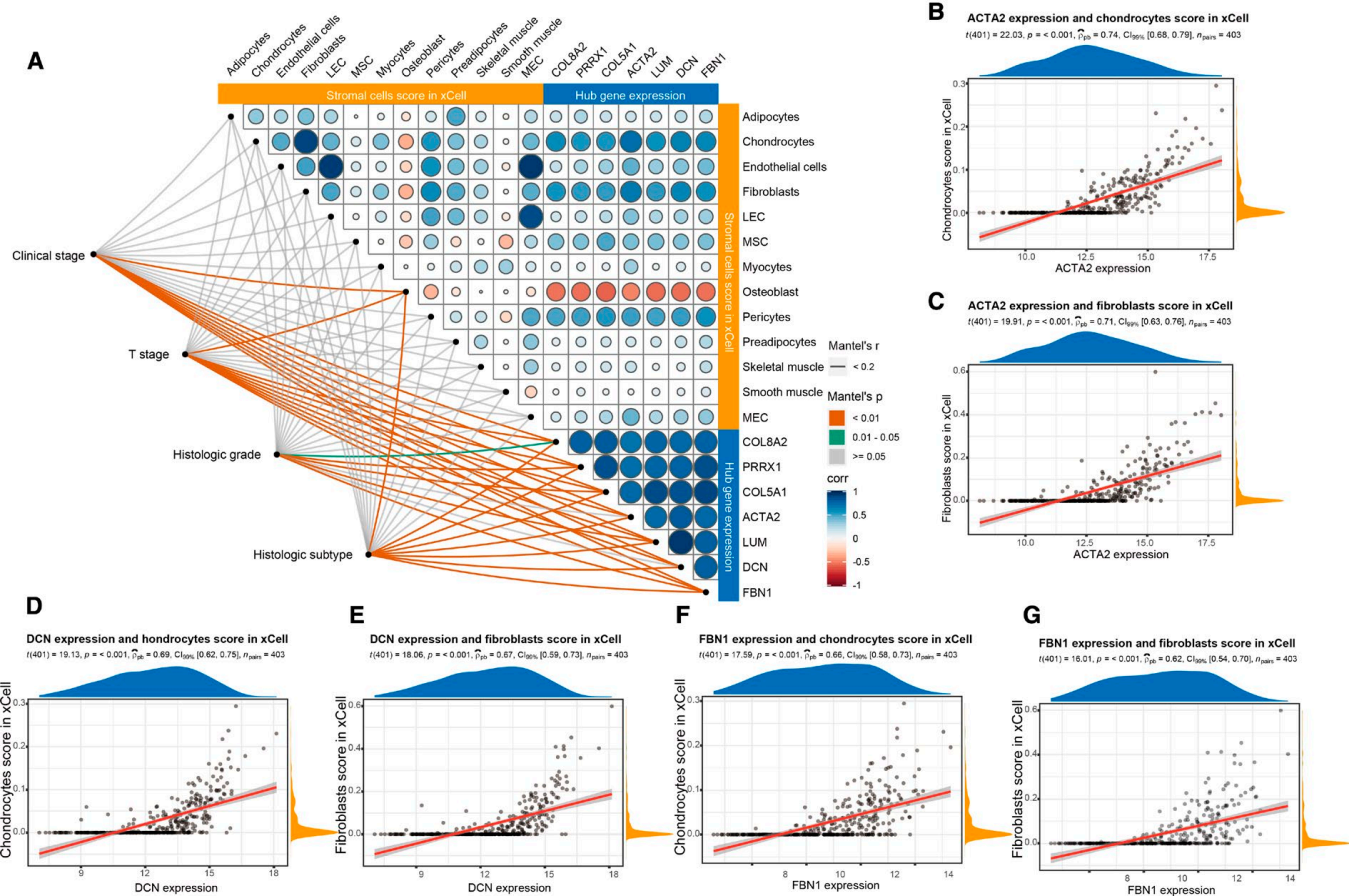
Correlation analysis of hub genes expression, different stromal cells score in xCell and clinical phenotype of MIBC. A, The expression of hub genes was correlated with the enrichment score of various stromal cells and clinical phenotype in MIBC. The size and color of the points were related to the correlation. The line color was related to the *P* value. B‐G, The expression of *ACTA2*, *DCN*, and *FBN1* were significantly correlated with the enrichment score of chondrocytes and fibroblasts (cor > .6, *P* < .001). LEC, lymphatic endothelial cells; MEC, microvascular endothelial cells; MIBC, muscle‐invasive bladder cancer; MSC, mesenchymal stem cells

The correlation between the expression of hub genes and the enrichment score of stromal cells in tissue samples was analyzed. It was found that the expression of hub genes was positively correlated with the enrichment score of stromal cells (except for osteoblast) and clinical phenotype (Figure 8A). The expressions of *ACTA2, DCN,* and *FBN1* were positively correlated with the enrichment score of chondrocytes and phagocytes (cor > .6, *P* < .001). (Figure 8B‐G).

### 3.9 The expression of hub genes was closely related to tumor invasion

**FIGURE 9:**
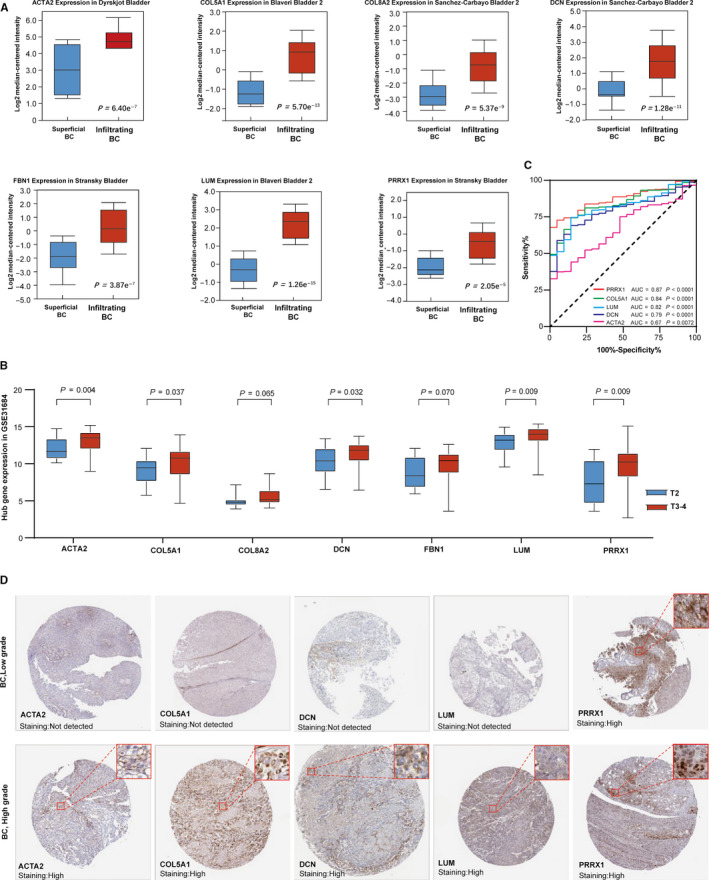
Verify the relationship between hub genes expression and tumor aggressiveness. A, Hub genes were expressed in infiltrating BC and superficial BC in the Oncomine database. B, Hub genes were expressed in MIBC of different T stages in the GEO database. C, The ROC curves of prognostic models based on histologic grade. D, IHC of hub genes at the different histologic grades of BC. AUC, area under the curve; BC, bladder cancer; GEO, gene expression omnibus; IHC, Immunohistochemistry; MIBC, muscle‐invasive bladder cancer; ROC, receiver operating characteristic; TCGA, The Cancer Genome Atlas

We found that seven key genes were significantly higher in infiltrating BC than in superficial BC use the Oncomine database. Its high expression means that the tumor was more aggressive (Figure 9A). In order to verify the expression levels of seven hub genes related to prognosis in MIBC, GSE13684 chip data was obtained from the GEO database. The results showed that the expression of five hub genes, including *ACTA2, COL5A1, DCN, LUM*, and *PRRX1*, were statistically significant at high T stage (T_3‐4_) and low T stage (T_2_) (*P* < .05). (Figure 9B). To further verify the diagnostic role of hub genes in different aggressive MIBCs, the ROC curve analysis and immunohistochemistry based on the results of THE HUMAN PROTEIN ATLAS were performed on five hub genes that were differentially expressed after validation in the GEO database. The results showed the four hub genes (*ACTA2, COL5A1, DCN, LUM*) had diagnostic value for high and low grade MIBC (Figure 9C, D). *PPRX1* showed diagnostic significance for differentiating high and low grade MIBC in the ROC curve, but the difference was not obvious in immunohistochemical staining. (Figure 9D).

## 4 DISCUSSIONS

Stromal cells in TME are closely related to the malignancy of tumors and can enhance tumor invasion, metastasis, and tumor angiogenesis.^24^ In this study, we used the ESTIMATE method to perform stromal score on stromal infiltration of tumor samples from 403 MIBC patients and to analyze the differences among samples with different clinical characteristics. The results confirm that stromal cells infiltration is significantly correlated with MIBC progression and that patients with high stromal cells infiltration in tumor samples have worse overall survival time.

In this study, we not only found that the stromal cells in the TME promoted the progress of MIBC and were mainly involved in the biological processes such as cell migration, leukocyte activation, and other signaling pathways such as PI3K‐Akt, MAPK, and Rap1, but also identified five stromal cells related to prognosis. Among them, chondrocytes and fibroblasts as the main stromal cell types of TME. Chondrocytes secrete the uPA/uPAR complex, which is an important component of various inflammation, and it can activate the PI3K‐Akt signaling pathway, induce the release of type I collagen by macrophages, and then promote tumor progression through α2β1.^25^, ^26^ The downstream mTOR signal can also regulate the epithelial‐mesenchymal transition (EMT) to promote the development of BC.^27^, ^28^ Cancer‐associated fibroblasts (CAFs) can induce the activation of neutrophils and avoid its apoptosis, while the activation of neutrophils can induce the formation of tumor blood vessels through the secretion of VEGFA, PROK2, and other vascular endothelial growth factors. Tumor proliferation can also be enhanced by secreting elastase to activate the PI3K‐Akt signaling pathway of the tumor.^29^, ^30^, ^31^ Besides, CAFs can induce monocytes in leukocytes to differentiate into M2‐like macrophages and activate lymphocytes, and then bind programmed death ligand 1 (PD‐L1) on the cell surface to its receptor PD‐1, and release anti‐T cell activation signals to suppress antitumor immunity and promote tumor progression,^32^, ^33^ Gok.^34^ Under the interaction between tumor cells and CAFs, p38αMAPK can induce the activation of phosphoglucomutase 1 in tumor cells, promoting glycolysis.^35^ This pathway can also regulate the expression of MMP‐2 and MMP‐9, which not only degrades the ECM, enhances the aggressiveness of tumor cells, but also maintains the microenvironment of tumor growth.^36^ Rap1 protein is one of the members of the Ras oncogene family. It transmits signals from tyrosine kinase receptors, G protein‐coupled receptors, and cytokine receptors, which regulates ERK/MAPK and other pathways, leading to cell proliferation, survival, and gene expression.^37^

Currently, some researchers have reported that hub genes such as *ACTA2, COL5A1, DCN, LUM*, and other genes are closely related to BC progression, but the relationship between hub genes and stromal cells in the TME has not been reported.^38^, ^39^, ^40^ In this study, we found that the expression of hub genes is significantly related to the ratio of chondrocytes and fibroblasts in the TME. The expression of *ACTA2* may stimulate the release of IL‐6 by CAFs to induce EMT and promote the progression of tumor phenotype from noninvasive to invasive phenotype.^41^ Its encoded cytoskeletal protein can interact with phosphoglycerate mutase 1, regulate the assembly of cellular actin filaments, control the contractile activity of tumor cells and fibroblasts, and regulate tumor metastasis.^42^, ^43^ Collagen encoded by *COL5A1* is associated with sports and connective tissue damage and is one of the main components of protein in CAFs.^44^, ^45^, ^46^ Bioinformatics has been predicted to be related to the occurrence and development of breast cancer, lung adenocarcinoma, renal clear cell carcinoma, and other tumors, but its role in BC has not been reported.^47^, ^48^, ^49^ The decorin expressed by the *DCN* gene is ubiquitous in the ECM and is a leucine‐rich proteoglycan (SLRP) secreted by stromal cells such as chondrocytes and fibroblasts. Decorin can activate the MAPK pathway by combining with epidermal growth factor, leading to the expression of downstream p21 genes related to tumor differentiation, invasion depth, hyperplasia and metastasis, and the value of prognosis.^50^, ^51^ The basement membrane glycan expressed by the *LUM* gene is another kind of SLRP, which can promote tumorigenesis by activating downstream FAK and MAPK pathways, but some studies have found that basement membrane proteins can also down‐regulate EGFR‐mediated Akt activity, leading to pancreatic ductal adenocarcinoma in vitro proliferation is reduced.^50^ The *PRRX1* product is a homologous domain transcription factor, which is upregulated by the key regulators of CAFs, thereby regulating the activation of fibroblasts.^52^ It is currently known as an EMT inducer, which can promote the migration and invasiveness of gastric cancer cells through the Wnt/β‐catenin pathway.^53^ The above analysis mainly reveals that these five genes regulate the invasion of MIBC through stromal cells, providing the potential for therapeutic targets.

In summary, this study analyzed the relationship between stromal cells and clinical phenotypes through stromal cells score and then performed bioinformatics analysis on the differential genes of samples with high and low stromal score. It was found that the biological role of DEGs mainly involves biological functions such as the activation of leukocytes and the signaling pathways such as PI3K‐Akt, MAPK, and Rap1. Screening five genes related to prognosis can be used as potential biomarkers for MIBC progress, which is helpful for targeted therapy of MIBC, and identified Chondrocytes and Fibroblasts as the main stromal cell types, guiding subsequent experimental research.

## CONFLICT OF INTEREST

The authors declare that the research was conducted in the absence of any commercial or financial relationships that could be construed as a potential conflict of interest.

## AUTHOR CONTRIBUTIONS

The study conception and design were performed by PL and JT. Material preparation, data collection, and analysis were performed by PL, JC, JL, ZY, DH, and LY. The first draft of the manuscript was written by PL, JC, and JL. The manuscript was revised by PL, ZW, and JT. All authors read and approved the final manuscript.

## Data Availability

Publicly available datasets were analyzed in this study, these can be found in The Cancer Genome Atlas (https://portal.gdc.cancer.gov), GEO database (https://www.ncbi.nlm.nih.gov/geo), cbioportal (http://www.cbioportal.org), ESTIMATE (http://bioinformatics.mdanderson.org/estimate), Oncomine database(https://www.oncomine.org/resource/main.html).
